# The current state and influencing factors of negative social expectations among thyroid cancer patients: a single-center cross-sectional analysis

**DOI:** 10.3389/fpsyg.2025.1599652

**Published:** 2025-05-12

**Authors:** Zhitong Wang, Cui Chen, Jing Lu, Zhenfan Liu

**Affiliations:** Department of Nursing, Deyang People's Hospital, Deyang, China

**Keywords:** thyroid cancer, negative social expectations, psychological resilience, loneliness, influencing factors

## Abstract

**Objective:**

To investigate the current status and influencing factors of negative social expectations in thyroid cancer patients, aiming to inform interventions that mitigate these expectations and foster positive psychological outcomes.

**Methods:**

From December 2022 to August 2023, we used convenience sampling to select 213 thyroid cancer patients who met the inclusion and exclusion criteria as research subjects. A questionnaire survey was conducted using a general information questionnaire, the Cancer patient Negative Social Expectation Scale, the simplified version of the psychological Resilience Scale, and the Cancer Loneliness Scale.

**Results:**

The median negative social expectation score among thyroid cancer patients was 10.00 (IQR: 6.00–17.50), with 31.0% of participants classified as having high negative social expectations. Univariate analysis revealed significant differences in negative social expectation scores across subgroups stratified by age, personality type, marital status, disease type (e.g., papillary vs. non-papillary carcinoma), and Insurance Type (*p* < 0.05). Correlation analysis demonstrated that the negative social expectation scores were inversely associated with psychological resilience (*r* =-0.426, *p* < 0.01) and positively correlated with the loneliness scores (*r* = 0.651, *p* < 0.01). Multiple linear regression analysis further identified psychological resilience (β = −0.32, *p* = 0.003), loneliness (β = 0.51, *p* < 0.001), disease type (papillary carcinoma vs. others; β = 0.18, *p* = 0.012), and Insurance Type (β = 0.15, *p* = 0.023) as significant predictors of negative social expectations, collectively explaining 50.70% of the total variance (*R*^2^ = 0.519).

**Conclusion:**

The negative social expectations of thyroid cancer patients are at a moderate level. patients with urban resident health insurance, non-papillary carcinoma subtypes, lower psychological resilience scores, and higher loneliness scores exhibited significantly elevated negative social expectation scores. To address this, targeted psychosocial interventions should be implemented for this population. These interventions aim to reduce negative social expectations, facilitate social reintegration, improve quality of life, and alleviate the socioeconomic burden on both families and society.

## 1 Introduction

Currently, with evolving disease patterns, thyroid cancer has emerged as one of the most prevalent endocrine malignancies (Cabanillas et al., 2016). In 2024, the Chinese National Cancer Center reported that thyroid cancer ranks third among all newly diagnosed cancers in China (Wang and Sun, 2024). The annual incidence of thyroid cancer is increasing at a rate of 20%, positioning it as the fastest-growing malignant tumor nationwide (Zhao et al., 2024). Despite its high incidence, thyroid cancer demonstrates relatively low recurrence and mortality rates compared to other malignancies, with a 10-year survival rate exceeding 90% (Yao et al., 2023). However, despite the favorable prognosis of most thyroid cancer patients, they still have more negative psychological and social barriers, which are often overlooked by clinicians (O'Neill et al., 2023). Some studies have confirmed that thyroid cancer is less malignant than other cancers, resulting in a common lack of effective psychological counseling and social support for them (Lin et al., 2024), Negative emotions including helplessness and loneliness have been reported among patients during early survivorship phases (Missaoui et al., 2022). Thyroid cancer survivor studies have also shown that due to social misconceptions and fear of cancer, changes in appearance due to surgical scarring, and endocrine effects of radioactive iodine therapy and hormone replacement therapy, about 60% of patients experience discomfort in their social life, negative emotions such as loneliness and anxiety, and negative social perceptions, which lead to a decline in the quality of survival (Chan et al., 2021; Giusti et al., 2020). Thus, negative psychology and negative social cognition of thyroid cancer patients have seriously affected their survival quality, but the current relevant studies mostly focus on the current status of survival, and do not pay enough attention to the impact and intervention of negative psychology and negative social cognition.

Negative social expectations (NSE), as a maladaptive psychological construct, describe the tendency of patients to interpret social interactions negatively when experiencing disease-related distress. Specifically, heightened negative emotions during illness increase vulnerability to perceiving ambiguous social cues as threatening, thereby fostering distorted beliefs about others' support intentions (Adams et al., 2017). When patients are diagnosed with cancer, their psychological fear increases abruptly and they are prone to develop an extremely strong social dependency psychology. When such dependency remains unmet, patients develop profound disillusionment with perceived social support deficits. This cognitive-emotional cascade amplifies social alienation, exacerbates anxiety and depressive symptoms, impairs treatment adherence, reduces quality of life, and correlates with elevated mortality risk in longitudinal studies (Lin et al., 2024). Chinese researchers have found that the negative social expectations of cancer patients are in the middle to upper level in the survey of survival status of cancer patients, which leads to the aggravation of their negative emotions and seriously affects their quality of life (Shang et al., 2023; Li et al., 2023). Notably, thyroid cancer patients were excluded from these analyses. preliminary data from thyroid cancer patients undergoing ^131^I isolation therapy indicate moderate NSE levels (*M* = 10.0, IQR = 6.0–17.5), which positively correlate with loneliness severity (*r* = 0.65, *p* < 0.01) (Fu, 2023). However, as ^131^I isolation represents a transient treatment phase, these findings cannot generalize to the broader thyroid cancer population. Collectively, NSE demonstrates robust interconnections with loneliness and quality of life across oncology populations. In order to reduce the negative psychology and negative social cognition of thyroid cancer patients, Chinese researchers should conduct a survey on the current status and influencing factors of negative social expectations in this population, and include psychological factors such as loneliness.

Loneliness, a prevalent negative emotional state among cancer patients, significantly compromises both mental health and quality of life (Adams et al., 2017). In thyroid cancer populations, approximately 30% of patients report clinically significant anxiety and depressive symptoms (Lam, 2022). Fu (2023) further corroborated moderate loneliness levels in this cohort, underscoring the critical need to address their psychosocial vulnerabilities. Empirical evidence demonstrates a robust negative correlation between psychological resilience and negative emotional states (*r* = -0.43, *p* < 0.01) in thyroid cancer patients (Xu et al., 2025). That is, the higher the level of psychological resilience, the more effectively patients can regulate their own emotions, reduce anxiety and depression levels, and thus improve their quality of life. Conversely, the lower the level of psychological resilience, the higher the level of negative emotions such as anxiety and depression, and the lower the quality of life of the patients.psychological resilience—defined as the capacity to adaptively confront adversity—serves as a protective factor against mental health deterioration (Xu et al., 2024). Cognitive-behavioral theory emphasizes the influence of individual thinking patterns on emotions and behaviors. Loneliness may lead to negative social cognition in patients, which may aggravate negative social expectations and survival pressure, and improving their psychological resilience can help them better cope with negative external evaluations and their own emotional distress, and reduce their loneliness and negative social expectations (Jiang et al., 2024). However, there are fewer studies on the correlation between psychological resilience, loneliness and negative social expectations of thyroid cancer patients, and there are limitations such as small sample size, lack of representativeness and single-center studies, which could not guide the clinical staff to carry out the relevant psychological treatment.

Building on this foundation, the present study examines the current status and influencing factors of negative social expectations (NSE) in thyroid cancer patients through the lens of cognitive behavioral theory. By integrating loneliness and psychological resilience into a multivariate analytical framework, this research aims to establish an empirical basis for developing targeted psychological interventions tailored to this population.

## 2 Methods

### 2.1 Participants

A convenience sampling method was employed to recruit thyroid cancer inpatients from Deyang people's Hospital (Sichuan province, China) between December 2022 and August 2023. Inclusion criteria: Diagnosis of differentiated thyroid cancer confirmed by histopathology (Ju et al., 2024); Full awareness of diagnosis with intact cognitive function; Age ≥ 18 years; provision of written informed consent. Exclusion criteria: Comorbidities involving other organ systems; History of additional malignancies; Sensory or communication impairments (hearing, vision, or speech).

Sample size estimation: Following the Kendall MG method, the required sample size was calculated as 5–10 times the number of questionnaire items (22 items), accounting for a 20% attrition rate and invalid responses. This yielded a target range of 132–264 participants. Ultimately, 213 patients were enrolled. Ethical approval was granted by the Institutional Review Board of Deyang people's Hospital (Approval No.: 2022-04-019-K01).

### 2.2 Methodology

#### 2.2.1 Survey instruments

##### 2.2.1.1 General information questionnaire

This self-administered questionnaire comprised 11 items assessing: Demographics: Age, gender, current residence, marital status, education level, Occupation Type; Clinical Characteristics: Disease location, disease type, duration of illness (months since diagnosis); Socioeconomic Factors: household economy, Insurance Type; psychological Traits: Self-reported personality type.

##### 2.2.1.2 Cancer negative social expectations scale (NSSESC)

The Negative Social Expectations Scale for Cancer (NSSESC) was originally developed by Adams et al. (2017) and cross-culturally adapted for Chinese populations by Cui and Sun (2020). This 5-item instrument employs a 6-point Likert scale (1 = strongly disagree to 6 = strongly agree), yielding total scores ranging from 5 to 30, with higher scores indicating greater severity of negative social expectations. psychometric properties: Demonstrated reliability in rectal cancer (Cronbach's α = 0.89; Sun et al., 2025) and nasopharyngeal carcinoma (α = 0.81; Li et al., 2023); Exhibited excellent internal consistency (α = 0.865) and content validity (Scale-level Content Validity Index/Average [S-CVI/Ave] = 0.97) in the current study.

##### 2.2.1.3 Simplified psychological resilience scale

The Simplified psychological Resilience Scale was culturally adapted and validated for Chinese cancer populations by Yu et al. (2011). This unidimensional scale comprises 10 items rated on a 5-point Likert scale ranging from never (0) to almost always (4), with total scores spanning 0–40. Higher total scores indicate stronger psychological resilience. In the current study, the scale demonstrated excellent reliability (Cronbach's α = 0.933).

##### 2.2.1.4 Cancer loneliness scale (CLS)

The Cancer Loneliness Scale (CLS) was originally developed by professor Adams at Stanford University (Adams et al., 2017) and cross-culturally adapted for Chinese populations by Cui and Sun (2020). This unidimensional scale comprises 7 items rated on a 5-point Likert scale ranging from never (1) to always (5), with total scores spanning 7–35. Higher scores reflect stronger perceived social isolation. psychometric properties in this study: Cronbach's α = 0.876 (CLS); Content validity: Scale-level Content Validity Index/Universal Agreement (S-CVI/UA) = 0.86;Average content validity: S-CVI/Average (S-CVI/Ave) = 0.98.

#### 2.2.2 Data collection methods

This study employed face-to-face questionnaire administration to ensure data accuracy and validity. The protocol included five key phases:

##### 2.2.2.1 Researcher training

All team members completed standardized training to ensure competency in: Guiding participants through questionnaire completion; Maintaining neutrality during data collection; Implementing quality control procedures.

##### 2.2.2.2 Participant recruitment

Subjects were consecutively enrolled from December 2022 to August 2023, with strict adherence to: predefined inclusion/exclusion criteria; Ethical protocols for informed consent; Sample representativeness optimization.

##### 2.2.2.3 Data collection protocol

For subjects with limited literacy ( ≤ 6 years of formal education): Items were verbally administered in Mandarin using lay terminology; Responses were simultaneously recorded via dual-channel backup: Digital audio recordings; paper-based documentation.

##### 2.2.2.4 Data verification

Two independent investigators: Transcribed audio recordings within 24 h of collection; Cross-validated transcriptions against paper records; Resolved discrepancies through consensus review.

##### 2.2.2.5 Response metrics

All the questionnaires were obtained from the research subjects, informed consent, a total of 230 questionnaires were distributed in this study, and 213 valid questionnaires were recovered, with an effective recovery rate of 92.6%.

#### 2.2.3 Statistical methods

Statistical analysis was performed using SpSS 25.0 software. Count data were described using frequency (%), and measurement data were described using median and interquartile range M (p25, p75). In univariate analysis, two-category comparisons used the Mann-Whitney U test, and multi-category comparisons used the Kruskal-Wallis H test; Spearman correlation analysis was conducted; and multiple linear regression analysis was performed for multifactor analysis. All data were subjected to two-sided tests, with a significance level of α = 0.05.

## 3 Results

### 3.1 General information patients with thyroid cancer

A total of 213 thyroid cancer patients were surveyed in this study, including 53 (24.9%) males and 160(75.1%) females; 53 (24.4%) rural residents and 161 (75.6%) urban residents; 177 (83.1%) married (refers to individuals in a legally recognized marriage) and 36 (16.9%) non-married (encompasses those who are single, divorced, or otherwise not currently married); 155 (72.8%) patients with papillary CA and 58 (27.2%) patients with non-papillary cancer.

### 3.2 Negative social expectations, loneliness, and psychological resilience scores of thyroid cancer patients

The results of this study showed that the total negative social expectation score for thyroid cancer patients was 10.00 (6.00, 17.50), the psychological resilience score was 30.00 (24.00, 37.00), and the loneliness score was 10.00 (8.00, 16.00). See Table 1.

**Table 1:** Negative social expectations, psychological resilience, and loneliness scores of thyroid cancer patients (*n* = 213).

**Item**	**Theoretical score**	**Actual score**
Total negative social expectation score	5 to 30	10.00 (6.00, 17.50)
Total psychological resilience score	0 to 40	30.00 (24.00, 37.00)
Total loneliness score	7 to 35	10.00 (8.00, 16.00)

### 3.3 Univariate analysis of negative social expectations in thyroid cancer patients

The univariate analysis revealed statistically significant differences (*p* < 0.05) in negative social expectation scores across the following demographic and clinical variables: age, marital status, personality, disease type, and Insurance Type. Detailed comparisons are presented in Table 2.

**Table 2:** General information and univariate analysis of Cr-NSES in thyroid cancer patients (*n* = 213).

**Item**	**Category**	**Number of examples**	**Negative social desirability M [p_25_,p_75_]**	**Statistical value (Z/H)**	***P***
Gender	Male	53 (24.9%)	12 (6.5, 17)	−0.775^a^	0.438
	Female	160 (75.1%)	10 (6, 18)		
Age	<30 years	31 (14.6%)	7.00 (5, 12)	10.242^b^	0.017
	31−40 years old	60 (28.2%)	11.5 (6, 19)		
	41–60 years	100 (46.9%)	12 (6, 17)		
	>60 years old	22 (10.3%)	7 (5, 17.25)		
Current residence	Countryside	52 (24.4%)	11 (6, 17)	−0.488^a^	0.625
	Cities and towns	161 (75.6%)	10 (6, 18)		
Marital status	married	177 (83.1%)	12 (7, 18)	−3.769^a^	0.000
	Unmarried	36 (16.9%)	6 (5, 9.75)		
Education level	Junior high school and below	72 (33.8%)	11 (6, 17)	0.382^b^	0.826
	Completed senior secondary education (academic/vocational track)	46 (21.6%)	9.5 (6, 17.25)		
	University and above	95 (44.6%)	11 (6, 18)		
Occupation type	Unemployed	50 (23.5%)	8 (5, 14)	6.461^b^	0.091
	Corporate/Institutional Employee	105 (49.3%)	12 (6, 18.5)		
	Freelance professional	32 (15%)	11 (5, 18.75)		
	Retired	26 (12.2%)	9 (6,17.25)		
Disease location	unilateral	135 (63.4%)	10 (6, 18)	−0.160^a^	0.873
	Bilateral	78 (36.6%)	10.5 (6, 17.25)		
Disease type	papillary CA	155 (72.8%)	10 (5, 17)	−2.100 ^a^	0.036
	Other CA	58 (27.2%)	12 (7, 19)		
Duration of illness	≤ June	87 (40.8%)	10 (6, 17)	0.384^b^	0.944
	6 months <and ≤ 1 year	46 (21.6%)	10.5 (6, 17)		
	1 year <and ≤ 3 years	57 (26.8%)	10 (5.5, 19)		
	More than 3 years	23 (10.8%)	11 (5, 16)		
Personality	Introverted	20 (9.4%)	6 (5, 10.75)	6.307^b^	0.043
	Extroverted	122 (57.3%)	12 (6, 18)		
	Intermediate type	71 (33.3%)	10 (6, 19.8)		
Insurance type	Urban employees	130 (61.0%)	8 (5, 14.25)	−4.358^a^	0.000
	Urban resident	83 (39.0%)	14 (8, 19)		
Household economy	≤ 5,000	80 (37.6%)	9.5 (6,18)	0.384^b^	0.944
	5,000 <and ≤ 10,000	86 (40.45%)	11 (6, 18.25)		
	More than 10,000	47 (22.1%)	10 (6, 16)		

a = Z-value, b = H-value.

### 3.4 Correlation analysis of negative social expectations in thyroid cancer patients

Spearman correlation analysis results showed that the negative social expectation score of thyroid cancer patients was negatively correlated with the psychological resilience score (*r* = −0.426, *p* < 0.01) and positively correlated with the loneliness score (*r* = 0.651, *p* < 0.01); psychological resilience was negatively correlated with loneliness score (*r* = −0.379, *p* < 0.01).

### 3.5 Multiple regression analysis of influencing factors of negative social expectations in thyroid cancer

A multiple linear regression analysis was conducted to identify predictors of negative social expectations in thyroid cancer patients, with the total negative social expectation score as the dependent variable. Independent variables encompassed demographic factors (age, marital status [0 = unmarried, 1 = married], personality type), disease type (1 = papillary carcinoma, 0 = Other carcinoma), socioeconomic factors (Insurance TypeCategories), and psychosocial measures (psychological resilience and loneliness scores), with detailed variable coding provided in Table 3. The analysis revealed four significant predictors (*p* < 0.05): non-papillary carcinoma histology, public health insurance coverage, elevated loneliness scores, and reduced psychological resilience. Complete regression coefficients are presented in Table 4.

**Table 3:** Variable assignment.

**Item**	**Assignment method**
(A person's) age	1 = ≤ age 30, 2 = 31 to age 40, 3 = 41 to age 60, 4 = > age 60
Marital status	1 = married, 2 = unmarried (divorced, widowed)
Personality	1 = introverted, 2 = extroverted, 3 = intermediate type
Disease type (whether papillary carcinoma)	1= Yes, 2 = No
Insurance type	1 = urban employees, 2 = urban resident
Psychological resilience	Raw score
Negative social expectations	Raw score
Loneliness	Raw score

**Table 4:** Multiple linear regression analysis of influencing factors of negative social expectations in thyroid cancer (*n* = 213).

**Independent variable**	**Regression coefficient β**	**Standard error (SE)**	**Standardized β^′^**	***t***	***P***	**95.0% CI**
(Constant)	2.503	2.602		0.962	0.337	
Loneliness	0.625	0.059	0.572	10.580	<0.001	0.509−0.742
Psychological resilience	−0.128	0.040	−0.170	−3.219	0.001	−0.206 to −0.050
(A person's) age	0.473	0.353	0.068	1.342	0.181	−0.222−1.169
Marital status	−0.862	0.835	−0.054	−1.032	0.303	−2.509−0.785
Personality	0.579	0.486	0.058	1.190	0.235	−0.380−1.538
Disease type (whether papillary carcinoma)	1.337	0.666	0.099	2.008	0.046	0.024−2.650
Insurance type	1.442	0.633	0.116	2.277	0.024	0.194−2.690

*R* = 0.721, *R*^2^ = 0.519, adjusted *R*^2^ = 0.503, *F* = 31.615, *p* = 0.000.

## 4 Discussion

### 4.1 Moderate level of negative social expectations in thyroid cancer

The results of this study showed that the total score of negative social expectation of thyroid cancer was 10.00 (6.00, 17.50), which indicated that the negative social expectation of thyroid cancer was at a moderate level, which was lower than that of Fu (2023) survey of patients with postoperative ^131^I isolation treatment for thyroid cancer and Shang et al. (2023) survey of patients with esophageal cancer (21.89± 3.53 points). The reasons for this are: (1) Compared to patients with postoperative ^131^I isolation treatment for thyroid cancer, more than 60% of the thyroid cancer patients in this study were in the early stages of treatment, with a disease duration of less than 1 year, had not yet received the physical discomfort caused by radiation and chemotherapy, and had a high self-survival ability and relatively little need for others, which led to lower negative social expectations. (2) Compared with esophageal cancer, thyroid cancer patients have a lower degree of malignancy, and the pressure of treatment and physical discomfort caused by cancer are relatively lighter, which may be the reason for the lower negative social expectations of the subjects in this study. (3) More than 60% of the participants in this survey had a high school education or higher, and they had a better understanding and were able to independently consult cancer-related knowledge, understanding that the recurrence and mortality rates of thyroid cancer were lower than those of other malignant tumors. Therefore, the cancer fear and negative emotions of the participants in this study were relatively low, and thus their level of negative social expectations was lower than that of patients with postoperative ^131^I isolation therapy for thyroid cancer and patients with esophageal cancer. The results of this study suggest that (1) medical personnel should strengthen thyroid screening to increase the early screening rate of thyroid cancer and reduce the cancer burden on patients (Wang and Sun, 2024). (2) Hospitals and the government should develop promotional policies to publicize thyroid cancer. For instance, healthcare institutions conduct monthly educational sessions on thyroid disorders and disseminate relevant knowledge via their WeChat public platforms. This will help increase public awareness and attention to the thyroid gland, reduce patients' psychological fear of thyroid cancer, and at the same time reduce the late detection rate of thyroid cancer.

### 4.2 Moderate level of loneliness in thyroid cancer patients

The results of this study show that the total loneliness score for thyroid cancer patients is 10.00 (8.00, 16.00), indicating that their loneliness is at a moderate level, and lower than the findings by Sun et al. (2025) for colorectal cancer patients (15.87 ± 6.12 points) and Zhang et al. (2024) for esophageal cancer radiotherapy patients (22.80 ± 3.72 points). This may be because, compared to colorectal and esophageal cancers, the treatment for thyroid cancer is primarily surgical, with lighter burdens from radiotherapy and chemotherapy, allowing most patients to return to society, resulting in less social dysfunction and psychological burden, and relatively lower loneliness. Additionally, in this study, 89.7% of patients were under 60 years old, enabling them to receive social support from family, friends, and society, alleviating the fear and loneliness brought about by cancer. Therefore, clinical healthcare professionals should emphasize the importance of social support from relatives and friends in interventions for loneliness in thyroid cancer patients, utilizing various forms such as mindfulness-guided emotional expression training, peer support education, and family empowerment education models to help patients establish more positive social expectations, thereby alleviating the negative impacts of loneliness (Verity et al., 2021; Lieberz et al., 2022). This not only helps improve individual mental health but also promotes better social integration.

### 4.3 Moderate level of psychological resilience in thyroid cancer patients

The results of this study showed that the total psychological resilience score for thyroid cancer was 30.00 (24.00, 37.00), indicating that the psychological resilience of thyroid cancer was at a moderately high level, which is in line with the survey of cervical cancer patients by Zhu and Li (2025) (31.27 ± 4.06). The Chinese government has clearly instructed in the “Guiding Opinions of the National Medical Security Bureau on the Establishment and Improvement of the “Dual-channel” Management Mechanism of National Health Insurance Negotiated Drugs” that special drugs such as chemotherapy and targeted drugs should be included in the scope of reimbursement of health insurance, which reduces the economic pressure of cancer patients, so that patients with thyroid and cervical cancers are able to maintain better psychological status and adaptability in the face of their illnesses. Psychological state and adaptability. In addition, 83.1% of the survey respondents are married, and 89.7% are under 60 years old. They were able to obtain family support from their husbands, parents and other multiple sources, thus reducing their negative emotions of loneliness and helplessness and enhancing their psychological resilience. Therefore, clinical staff should pay attention to the level of psychological resilience of thyroid cancer patients and carry out intensive training, such as Attention and Interpretation Therapy (AIT), to reduce cancer-related fatigue and negative emotions (Wang et al., 2024), in order to help patients cope with psychological stress caused by the disease more effectively (Ma et al., 2024).

### 4.4 Influencing factors of negative social expectations in thyroid cancer

#### 4.4.1 Disease type

Our results identify disease type as a significant predictor of negative social expectations in thyroid cancer patients (B = 1.337, *p* < 0.05). patients with papillary thyroid carcinoma demonstrated lower negative social expectations than those with non-papillary subtypes. Comparative analysis shows that (1) compared with non-papillary cancer, papillary thyroid cancer has mild symptoms, good prognosis, low recurrence and mortality rates, and shorter duration of radiotherapy in the later stages, which makes the patients have lower physical and mental fatigue and economic pressure than other types (Lin et al., 2024). Therefore, the disease burden of these patients is relatively low. (2) Non-papillary thyroid cancer is highly malignant, poorly treated and has a long treatment period, which puts a double burden on patients psychologically and economically. Their need for social support is also increased, and once it is not satisfied, they are very likely to have negative social expectations. Therefore, clinical work should pay more attention to the negative social expectations of patients with non-papillary thyroid cancer, and psychological interventions such as cognitive behavioral therapy (CBT) and group therapy should be carried out for this group of patients to provide timely and appropriate social support to help them overcome the fear of the disease, cope with the pressure positively, and return to the society as soon as possible in order to improve the quality of life.

#### 4.4.2 Insurance type

In this study, insurance types were divided into urban employee patients and urban resident patients. The results indicate that insurance type is one of the main influencing factors of negative social expectations in thyroid cancer patients (B = 1.442, *p* < 0.05), with urban residents exhibiting higher negative social expectations. With the continuous improvement of China's medical insurance system, all survey subjects in this study had purchased medical insurance, providing relatively high socioeconomic support, which is one reason why the results of this survey are lower than previous studies. Additionally, the “Notice on Doing a Good Job in Basic Medical Security for Urban and Rural Residents in 2025” clearly stipulates different reimbursement ratios for different insurance types, with urban residents generally having lower reimbursement ratios (50–80%) compared to urban employees (75–95%). Consequently, urban resident patients bear relatively greater economic pressure. Furthermore, urban resident patients often consist of unemployed individuals, self-employed individuals, or farmers with relatively low incomes, further increasing their economic burden of treatment and leading to negative social cognition and higher levels of negative social expectations. Therefore, clinical healthcare professionals should pay attention to the economic pressures brought about by insurance types in psychological interventions for thyroid cancer patients, implementing corresponding measures to alleviate their economic burdens. For example, based on the relatively low late-stage treatment and good prognosis of thyroid cancer, healthcare professionals can compare treatment costs with those of other cancers, explaining the treatment plans for thyroid cancer to help patients face economic pressures and enhance their confidence in participating in treatment.

#### 4.4.3 Loneliness

The results of this study show that loneliness levels are an influencing factor for negative social expectations in thyroid cancer patients (B = 0.625, *p* < 0.001). In predicting negative social expectations in thyroid cancer patients, loneliness has a positive effect, meaning that the lower the loneliness level, the lower the negative social expectations; conversely, the higher the loneliness level, the higher the negative social expectations. This is consistent with the findings of Shang et al. (2023), possibly related to the perception in Chinese culture that cancer patients are often seen as a burden to their families, and societal prejudice against cancer creates greater pressure for patients when seeking help and expressing emotions, leading them to endure not only physical suffering but also significant psychological stress. Compared to other diseases, cancer patients typically face dual pressures from the risk of cancer recurrence and high medical costs, increasing their desire for support from family and friends. When patients' expectations are unmet, they are likely to feel disappointed and isolated, triggering loneliness and depression. Therefore, medical staff should regularly assess the psychological status of cancer patients in their clinical work, and use individual psychological counseling and online psychological counseling to help them establish positive social expectations. At the same time, they should start from various aspects, such as family and friends, and use group support groups to improve the individual's social support network and mental health, so as to reduce the sense of loneliness and negative social expectations (Wang et al., 2024), and break the vicious cycle of loneliness (Torres et al., 2024).

#### 4.4.4 Psychological resilience

The results of this study indicate that psychological resilience is one of the main influencing factors of negative social expectations in thyroid cancer patients (B = −0.128, *p* ≤ 0.01). The higher the psychological resilience score of thyroid cancer patients, the lower their negative social expectation score, consistent with the findings of Zhang et al. (2024). Psychological resilience determines whether individuals can timely adjust their mindset and cope positively when faced with trauma or setbacks. Patients with high levels of psychological resilience can self-regulate, release negative emotions, and face the pressures of disease and life more actively, facilitating their reintegration into society. Therefore, when treating patients with thyroid cancer, medical personnel should emphasize the assessment and enhancement of psychological resilience, and combine physiological and psychological interventions in order to improve the overall health and quality of life of patients. First, medical personnel can improve the psychological resilience of patients through various methods, such as positive psychology counseling and positive psychology cultivation, to help them manage cancer-related stress and negative emotions more effectively and reduce negative social expectations, so as to adapt to social life more quickly (Ma et al., 2024). Second, for patients with low psychological resilience, medical staff can promote their psychological resilience by implementing personalized psychological counseling and behavioral interventions. These interventions can help patients better cope with the disease and also reduce the burden on their families and society, thus forming a benign support system.

## 5 Conclusion

In summary, the negative social expectations of thyroid cancer patients were at a medium level, and the negative social expectations of patients with disease classification of non-papillary cancer, urban resident insurance status, higher loneliness score and lower psychological Resilience score were heavier. Therefore, in clinical work, medical personnel not only need to pay attention to the effect of cancer treatment, but also need to carry out the intervention of their negative social expectations. First of all, medical personnel need to reduce the negative social expectations of thyroid cancer patients through psychological intervention and the establishment of social support networks. By educating patients to face up to the disease and the social support of others, they can help them to reduce their negative views of their condition and thus improve their psychological state. For example, organizing support groups or psychological counseling can effectively alleviate patients' sense of isolation and enhance psychological resilience. Secondly, medical staff should actively guide patients to participate in social activities and encourage them to have positive interactions with their families and friends. This not only reduces negative social interactions, but also enhances patients' sense of social belonging, helps them return to society as soon as possible, and reduces family and social pressure. This positive sense of participation helps to improve patients' quality of life and enhance their adherence to treatment. Lastly, hospitals should step up screening for thyroid nodules in order to reduce the incidence of thyroid cancer. Society and the government should increase financial support for cancer patients, such as establishing a cancer fund. By doing so, the mental health and social support of patients can be enhanced, which will in turn reduce negative social expectations and improve the overall quality of life of patients.

## 6 Limitations and prospects

This study has the following limitations due to factors such as manpower and time: (1) This is a single-center survey that included only 213 patients with thyroid cancer, most of whom had a disease duration of less than 1 year, resulting in a small sample size and insufficient representativeness. (2) This study only included patients with differentiated thyroid cancer (excluding other rare forms such as poorly differentiated and undifferentiated thyroid cancer), and all subjects were Chinese patients, which limits the scope, culture, and national context of the research. (3) This study is a cross-sectional survey and cannot capture changes in social expectations at different stages of the disease. (4) This study only explored the correlation between certain demographic information such as age and gender, psychological resilience, loneliness, and negative social expectations, which limits the depth and breadth of the research. Therefore, the next step will be to expand the sample size and conduct longitudinal studies on negative social expectations based on different disease types of thyroid cancer across multiple centers and regions, exploring the trajectory of negative social expectations at different types and stages of the disease. Additionally, we will incorporate individual factors such as self-efficacy and personality traits, as well as external factors like social support and environmental stress, to enhance the depth and breadth of the research. Through continuous exploration, we aim to provide a reference for intervention studies on negative social expectations in thyroid cancer patients, helping them better cope with the challenges posed by cancer and improve their quality of life.

## Data Availability

The raw data supporting the conclusions of this article will be made available by the authors, without undue reservation.
