# Supplementary material for: The current state and influencing factors of negative social expectations among thyroid cancer patients: a single-center cross-sectional analysis
Source: Front Psychol. 2025 May 12;16:1599652. doi: 10.3389/fpsyg.2025.1599652 (PMC12104241; doi:10.3389/fpsyg.2025.1599652)
Supplement: Supplementary file 1 [file Supplementary_file_1.docx]

| **Table S1 Cancer Negative Social Expectations Scale (NSSESC)** | | | | | | |
| --- | --- | --- | --- | --- | --- | --- |
| **Item** | **Strongly disagree** | **Moderately disagree** | **Slightly disagree** | **Slightly agree** | **Moderately agree** | **Strongly agree** |
| If I disclose my diagnosis to others, they would feel uncomfortable being around me. |  |  |  |  |  |  |
| If people avoid discussing my condition with me, I would interpret it as their unwillingness to listen. |  |  |  |  |  |  |
| People cannot truly understand the emotional experience of living with this illness. |  |  |  |  |  |  |
| Sharing my illness-related thoughts and feelings with others would create an undue psychological burden on them. |  |  |  |  |  |  |
| Since being diagnosed with this condition, others fail to comprehend my existential concerns about the future. |  |  |  |  |  |  |
| This 5-item instrument employs a 6-point Likert scale (1 = strongly disagree to 6 = strongly agree), yielding total scores ranging from 5 to 30, with higher scores indicating greater severity of negative social expectations. | | | | | | |

| **Table S2 Simplified psychological Resilience Scale** | | | | | |
| --- | --- | --- | --- | --- | --- |
| **Item** | **Never** | **Rarely** | **Sometimes** | **Often** | **Always** |
| 1.When things change, I can adapt. |  |  |  |  |  |
| 2.I can cope with whatever happens in life. |  |  |  |  |  |
| 3.When facing difficulties, I try to focus on the positive aspects. |  |  |  |  |  |
| 4.Going through hardships makes me stronger. |  |  |  |  |  |
| 5.I recover easily from illnesses, injuries, or setbacks. |  |  |  |  |  |
| 6.I believe I can achieve my goals even with obstacles. |  |  |  |  |  |
| 7.I can think clearly and stay focused under pressure. |  |  |  |  |  |
| 8.I don’t let failures defeat me easily. |  |  |  |  |  |
| 9.I consider myself strong in dealing with life’s failures and challenges. |  |  |  |  |  |
| 10.I can manage unpleasant or painful emotions, such as sadness, fear, and anger |  |  |  |  |  |
| This unidimensional scale comprises 10 items rated on a 5-point Likert scale ranging from never (0) to almost always (4), with total scores spanning 0–40. Higher total scores indicate stronger psychological resilience. | | | | | |

| **Table S3 Cancer Loneliness Scale (CLS)** | | | | | |
| --- | --- | --- | --- | --- | --- |
| **Item** | **Never** | **Rarely** | **Sometimes** | **Often** | **Always** |
| Since being diagnosed with the illness, do you feel that even your closest friends and family cannot understand you? |  |  |  |  |  |
| Do you feel that others cannot provide the support you need to cope with the illness? |  |  |  |  |  |
| Since the diagnosis, do you feel different from people around you? |  |  |  |  |  |
| Do you feel unable to talk to anyone about your thoughts and feelings regarding the illness? |  |  |  |  |  |
| Since being diagnosed, do you feel unneeded by others? |  |  |  |  |  |
| Since the diagnosis, do you feel empty inside? |  |  |  |  |  |
| Does the illness make you feel cut off from others? |  |  |  |  |  |
| This unidimensional scale comprises 7 items rated on a 5-point Likert scale ranging from never (1) to always (5), with total scores spanning 7–35. Higher scores reflect stronger perceived social isolation. | | | | | |
